# Observations on Medical Education

**Published:** 1813-02

**Authors:** 


*To the Editors of the Medical and Physical Journal.*

GENTLEMEN,

THE great complaint of so many medical writers, of the want of adequate compensation for their labor, and the lamentations of the London Apothecaries on the advanced price of vials and corks, has induced me to make some inquiries into the state of the matter.

I am given to understand, that the demand which government make annually for a supply of surgeons for the army and navy, and the constant employment of men-midwives in the country, are the true causes why the education towards surgery becomes so superficial, and why the practice of it in the country is so deficient.

I do not pretend to say that the examination of the College of Surgeons is imperfect; they have *orders* to pass a certain number, monthly, for the service of government; and, if they are over-nice, our vessels and regiments must be incomplete: it is assistants they are in want of, not surgeons; and, if those young gentlemen were examined practically as to the application of a torniquet or a bag truss, the cure of an ophthalmia or a gonorrhœa, the preparations necessary to be made previous to the performance of a grand operation, or any of those requisite duties which should be executed before and after the bloody scene of the tragedy, they would be obliged to send back two-thirds of the applicants, and then what would become of the funds for upholding such a magnificent building, or how would the Courts of Assistants and Examiners be able to feed so luxuriously?

The College, therefore, must necessarily pass a vast number of persons; and, such persons having passed, have, from a right of service, the privilege of settling wherever they please, please, can write surgeon over their door, and, at an under price, practise midwifery. The women are taught to believe that midwifery is a divine art, and, as long as it is but a man, they are willing to let the wisest and quietest old lady in the parish stay at home and starve for want of employment.

Now how can it be expected, that a tolerably well-informed surgeon, apothecary, and man-midwife, who is five nights in the week galloping across the commons all night, and home the next day; how can it be expected, I say, that such a sleepless practitioner should be much awake the next morning to follow up his pharmacy and surgery? He takes an apprentice for the sake of the premium, but in five years service does he teach this apprentice any-thing of his art and mystery? Does he employ him in any-thing but making up draughts, writing labels, and, at Christmas, writing out the bills?

Now, was he to give him, as he is bound to do, one lesson a week in the art and mystery of surgery and medicine, it would be no hard task to give or to learn; but it would be fifty-two lessons in the first year, which would make any but a sad blockhead mechanically clever; it would induce a lad to love his profession and his master, and it would make him a valuable servant for the rest of his period of service; it would send him to the London hospitals with an understanding to comprehend what he saw, and the lectures he heard; it would prepare him to finish an education which had began properly, and to be adequately rewarded because he would be able to cure. Every peasant who needs advice is willing to pay for it, if it serves his purpose; but he grudges to pay a long bill for *stuff*, when neither he or his dame are one doit the better

Certainly, Gentlemen, there are too many bad surgeons every where, and not enough good ones. I do not style that man a good surgeon who is galloping over a country to look for a femoral hernia, or a disease of the arterial system, that he may shew his dexterity by cutting down on a blood-vessel; it is not the anatomical dissector alone that makes the surgeon. He is the best surgeon, in my opinion, who has the least use for his instruments, who can prevent or cure a disease without maiming or defacing.

I have a nephew in the profession, who assures me, that the well-informed practitioners make no complaints, nay, even that they do not object to a little regular quackery, for mankind love to be quacked, as is evident in other matters as well as in medicine. He evinces the Eau Medicinale, and declares some of the best and most efficacious medicines in in the Pharmacopœia of the London College, are taken from those who have been esteemed empirics.

Let the faculty of surgeons then amend themselves; let no lecturer be allowed to admit a pupil who does not bring with him credentials; let every master do his duty by his apprentice; and, though the number be lessened, there will yet be enough for the use of mankind: the undertakers will have us all in good time, but some of us may live to see better times, and pay our Christmas bills with thankfulness for health restored. I am, &c.

SENEX.

